# Validation of the Japanese Version of Obstetric Quality of Recovery-11 Questionnaire and Its Association with Postpartum Depression and Functional Outcomes: A Prospective Observational Study

**DOI:** 10.3390/jcm14041390

**Published:** 2025-02-19

**Authors:** Ayu Ishida, Mitsuru Ida, Yusuke Naito, Akane Kinomoto, Masahiko Kawaguchi

**Affiliations:** 1 Department of Perioperative Management Centre, Nara Medical University Hospital, Kashihara Shijo 840, Nara 634-8522, Japan; k185576@naramed-u.ac.jp; 2 Department of Anesthesiology, Nara Medical University, Kashihara Shijo 840, Nara 634-8522, Japan; naito623@naramed-u.ac.jp (Y.N.); drjkawa@naramed-u.ac.jp (M.K.)

**Keywords:** cesarean delivery, patient-reported outcome measures, patient safety, perioperative medicine

## Abstract

**Background/Objectives**: The aim was to develop a Japanese version of the Obstetric Quality of Recovery-11 questionnaire (ObsQoR-11J), assess its feasibility, reliability, and validity, and investigate its association with postpartum depression and functionality. The need for this study is underscored by the limited availability of the ObsQoR-11 in different languages and the lack of documentation on its associations with early postpartum recovery and mid-term postpartum patient-reported outcomes. **Methods**: After translating the ObsQoR-11J into Japanese, 138 patients who underwent non-emergent cesarean delivery were enrolled in this study. ObsQoR-11J scores were evaluated at 24 h, 3 days, and 5 days post-surgery. The associations between ObsQoR-11J scores and postpartum depression and functionality, which were assessed using the Edinburgh Postnatal Depression Scale (EPDS) at 1 and 3 months and the 12-item World Health Organization Disability Assessment Schedule (WHODAS) 2.0, respectively, at three months after cesarean delivery, were evaluated. **Results**: The questionnaire completion rate at 24 h was 97.1% (134/138), and the mean ObsQoR-11 scores at 24 h and 3 and 5 days post-surgery were 67.2, 89.0, and 96.3, respectively. Cronbach’s alpha was 0.77, and the Spearman correlation coefficient between ObsQoR-11J scores and global health visual analog scale scores was 0.43 (*p* = 0.03) at 24 h. The ObsQoR-11 score at any measurement point was significantly associated with the EPDS and 12-item WHODAS2.0 after adjusting for clinically relevant factors (all *p* < 0.05). **Conclusions**: The ObsQoR-11J is a valid assessment tool, and its scores are associated with patient-reported outcome measures.

## 1. Introduction

Postoperative recovery is no longer measured with mortality and morbidity outcomes but with a more dynamic understanding of the patient experience and quality of recovery from surgery. For example, interest in outcomes immediately after surgery has shifted from traditional outcomes such as pain scores and opioid consumption to more multidimensional tools for the assessment of post-cesarean functional recovery.

The Quality of Recovery (QoR) questionnaire is the most widely used outcome measure for early and immediate postoperative functional recovery [1,2]. However, the QoR questionnaire was developed to assess postoperative recovery in non-obstetric populations; thus, the obstetric QoR-11 (ObsQoR-11) questionnaire was introduced in 2019, allowing for the assessment of recovery after giving birth [3]. Although the ObsQoR-11 has been translated into several languages, the Japanese version of the ObsQoR-11 (ObsQoR-11J) is not available. In addition, their ability to meet certain critical milestones, such as mid-term functional and mental recovery after surgery, has been unknown [4,5,6].

Postpartum depression may delay breastfeeding initiation, disrupt bonding between the mother and infant, and increase maternal suicide risk [7,8]. However, postpartum maternal mental health and functioning remain poorly documented [9,10]. Previous studies have shown that poor recovery in hospitals after cardiac and abdominal cancer surgery can be a predictor of poor quality of life and functional status three months thereafter [11,12]. However, little evidence is available regarding the association between short-term outcomes, such as ObsQoR-11 scores, and mid-term outcomes, including mental health and functioning. One prospective observational study conducted in a single center in the Middle East revealed that poor inpatient postpartum recovery was associated with postpartum depression at six weeks [13]; however, further studies are required to validate these findings.

This study aimed to develop a valid version of the ObsQoR-11J and evaluate its feasibility, reliability, and validity in patients who underwent cesarean delivery under spinal anesthesia. We also aimed to explore the association between the ObsQoR-11J and postpartum mental health and functioning.

## 2. Materials and Methods

This prospective observational study was approved by the Institutional Review Board of Nara Medical University (approval number: 3051; 9 March 2023). Patients who were scheduled for cesarean delivery were informed about this study the day before surgery, and those who underwent urgent cesarean delivery were informed about this study when the decision was made to have a cesarean delivery. Written informed consent was obtained from all the patients before enrolment. This study was conducted in accordance with the 2013 Declaration of Helsinki and followed the Strengthening the Reporting of Observational Studies in Epidemiology (STROBE) guidelines.

### 2.1. Patient Selection

This prospective study, conducted at Nara Medical University between April 2023 and January 2024, included patients aged ≥18 years who underwent non-emergency cesarean delivery under spinal anesthesia [14], which was predicted to begin after 8:30 am and end by 7:00 pm owing to staff resources. Patients who attended psychiatry and received drug treatment who were not fluent in Japanese underwent cesarean delivery with interventions other than single-dose spinal anesthesia, did not have access to email, or delivered a fetus not expected to survive were not eligible for this study. Patients who refused to participate were excluded from this study. Patients undergoing cesarean delivery under spinal anesthesia at our hospital received 0.5% hyperbaric bupivacaine (10–15 mg), morphine hydrochloride (100 µg), and fentanyl (10 µg) in the subarachnoid space. Postoperative analgesia was provided using acetaminophen (1000 mg for ≥50 kg, 15 mg/kg for <50 kg) administered every 6 h for up to 4 doses 24 h after surgery upon return to the obstetric ward. Subsequently, loxoprofen, sodium hydrate, and/or oral acetaminophen were prescribed according to the patient’s symptoms. None of the patients were prescribed opioids postoperatively.

### 2.2. Development of the ObsQoR-11J

The ObsQoR-11, a specific measurement tool used to evaluate recovery after delivering a baby, consists of 11 items, including maternal recovery and the ability to care for an infant [3]. For each item, the patient was assigned a score from 0 to 10 points to describe their state; thus, the total score ranged from 0 to 110 points, with higher scores indicating better recovery. After obtaining permission from the original authors (Ciechanowicz et al. [3]) on April 8, 2019, the ObsQoR-11 was translated according to the methods adopted by the International Quality of Life Assessment Project [15].

Two doctors (M.I. and Y.N.) independently performed the forward translation from English to Japanese, and the two translations were merged into a preliminary version, which was assessed by A.K. and M.K. In the back-translation process, a translator fluent in Japanese and English and unfamiliar with the original version of the ObsQoR-11 translated the ObsQoR-11 back into English. The questionnaire creators [3] evaluated each item to ensure consistency with the original content.

If a question differed from the original intent of the author, the translation and back-translation processes were repeated until the original author’s approval was obtained. Ten patients were selected with permission from the original author to ensure that all items in the ObsQoR-11J were easily understood and answered. All participants responded to each question without difficulty, and the final version of the ObsQoR-11J was completed (Table S1).

### 2.3. Patients’ Characteristics and Perioperative Data

Before surgery, we recorded the patient’s age, height, weight, alcohol consumption, smoking status, gestational age, presence of a partner, cesarean delivery history, infertility treatment history, anxiety and depression, gravidity, number of fetuses, comorbidities (hypertensive disorders of pregnancy, gestational diabetes, and thyroid disease), ritodrine administration, magnesium infusion, and urgent surgery indications. After surgery, surgical duration and intraoperative blood loss were recorded. Anxiety and depression levels were assessed using the Hospital Anxiety and Depression Scale [16], which is a self-assessment tool consisting of 14 items, with 7 items each for the anxiety and depression subscales, with each item scored between 0 and 3 points. A total score of ≥8 points indicated positive screening for anxiety and depression [16].

### 2.4. Outcomes

The primary outcome in this study was the ObsQoR-11J score, which was assessed at three postoperative points through e-mail correspondence: 24 h, 3 days, and 5 days after cesarean delivery. Moreover, 20 patients completed the ObsQoR-11J at 25 h to evaluate the reliability of the responses. The secondary outcomes in this study were the Edinburgh Postnatal Depression Scale (EPDS), which was assessed 1 and 3 months after cesarean delivery, and the 12-item World Health Organization Disability Assessment Schedule (WHODAS) 2.0 [11,12,17]. This score was assessed three months after cesarean delivery.

The EPDS is a questionnaire containing ten items answered by mothers and is widely used to screen for postpartum depression [18]. Each question was assigned a score of 0–3 points for a total score of 0–30 points, with higher scores indicating higher depressive tendencies [18]. The cutoff value for the Japanese population was 9 points [19].

The 12-item WHODAS 2.0 has a total score of 0 (no disability) to 48 (full disability). This score has been validated for postpartum women [10,20]. For clinical use, the total score was converted into a percentage [17]. Each questionnaire was sent by e-mail. If there was no response, the researcher contacted the patient via the telephone. If there was no response after two attempts, the case was classified as non-response. Survival with a WHODAS score < 16% was defined as disability-free survival [21]. Several previous studies have used EuroQol 5 dimensions 3-level (EQ-5D-3L) as a health indicator immediately after cesarean section [22,23,24]; however, this indicator may not be suitable as a mid-term postoperative measure because it is assessed on the same day that it is administered. In addition, different countries have different interpretations of the same EQ-5D-3L value; thus, simple comparisons between countries are not possible [25]. Thus, we used the WHODAS2.0 because the American Society of Anesthesiology recommends WHODAS 2.0 as a patient-reported outcome measure after hospital discharge, although it is unclear whether this statement reflects the above facts [1].

### 2.5. Statistical Analysis

Continuous and categorical variables were presented as mean ± standard deviation and count (%), respectively.

Feasibility was assessed using the patient recruitment rate, successful completion rate, floor effect, and ceiling effect 24 h after cesarean delivery. Patient recruitment and successful completion rates were calculated by dividing the relevant number of patients by the total number of patients. The floor and ceiling effects were assessed as the percentage of patients with the lowest and highest scores, respectively. Reliability was assessed using Cronbach’s alpha (internal consistency) and test–retest reliability (*n* = 20). Cronbach’s alpha was >0.70, which is considered acceptable in the clinical setting [26]. Test–retest reliability was assessed using the Spearman correlation coefficient between ObsQoR-11J scores at 24 and 25 h after cesarean delivery. Validity was assessed using the Spearman correlation coefficient between the ObsQoR-11J and the global health visual analog scale scores and inter-item correlations. The change in ObsQoR-11J score until postoperative day five was assessed using a linear mixed model, and the time points were treated as categorical variables. The association of ObsQoR-11J with secondary outcomes was assessed using Spearman correlation and multiple linear regression analyses, adjusting for clinically relevant factors, such as age, urgency, presence of a partner, infertility treatment history, preoperative anxiety or depression, and surgical duration. Multiple imputations for missing data are not provided.

Based on the guidelines that recommend at least 10 subjects per item on the instrument scale, the minimum required sample size was calculated as 110 patients, as the ObsQoR-11J includes 11 items [26]. Considering a 20% dropout rate, we aimed to enroll 138 patients, which is the sample size required for multiple regression analysis involving seven covariates. All statistical analyses were performed using SPSS version 25.0 (IBM, Armonk, NY, USA). Statistical significance was set at *p* < 0.05.

As a post-hoc analysis requested during the peer review process, we calculated the values of Cronbach’s coefficient for EPDS at 1 month and 3 months and for WHODAS. The values of Cronbach’s coefficient for EPDS at 1 month and 3 months and for WHODAS were 0.83, 0.74, and 0.87, respectively.

## 3. Results

**Figure 1:**
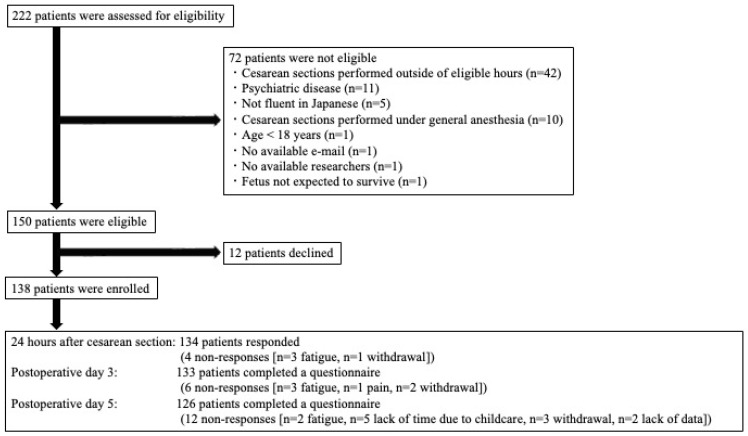
Patient flowchart.

Of 222 patients who underwent cesarean delivery, 72 were ineligible for this study. The most common reason for ineligibility was that cesarean sections were performed outside eligible hours (*n* = 42). Of the remaining 150, 12 patients declined; thus, 138 were included in this study. Four, six, and twelve patients had no response at 24 h, 3 days, and 5 days after cesarean delivery, respectively (Figure 1). The final analysis included data from 137 patients who had responded to the survey at least once. The patient characteristics are shown in Table 1.

The patient recruitment rate was 92.0% (138/150), and the successful evaluation completion rates were 97.1% (134/138), 95.6% (132/138), and 91.3% (126/138) at 24 h, 3 days, and 5 days after cesarean delivery, respectively. Each item had floor and ceiling effects 24 h after cesarean delivery; however, the total score did not have either floor or ceiling effects (Table 2). The mean time required to complete the ObsQoR-11J was 67 ± 38 s.

The ObsQoR-11 score 24 h after cesarean delivery showed a normal distribution (Figure S1). Cronbach’s alpha was 0.77, and the Spearman correlation coefficient for the test–retest was 0.43 (*p* = 0.03). The Spearman correlation coefficient between the ObsQoR-11J scores 24 h after cesarean delivery and the global health visual analog scale score was 0.60 (*p* < 0.001). The inter-item correlations are shown in Table S1. The mean ObsQoR-11 scores at 24 h and on postoperative days 3 and 5 were 67.2 ± 19.2, 89.0 ± 11.4, and 96.3 ± 9.6 points, respectively, and this score recovered over time (Table S2).

The mean EPDS scores at one month and three months after cesarean delivery were 4.5 ± 3.9 and 2.8 ± 2.7 points, respectively, and 16.1% (21/130) and 4.3% (5/115) of patients had positive screening for postpartum depression at one month and three months after cesarean delivery (Table 3). The mean weighted 12-item WHODAS2.0 score at three months after surgery obtained from 116 patients was 5.3 ± 8.1 points, and 86.3% (100/116) of the patients had disability-free survival (Table 3). The results of the Spearman correlation analysis are presented in Table S3. Significant associations were observed between the EPDS at one month after cesarean delivery and the weighted 12-item WHODAS2.0 score at three months after cesarean delivery (*p* < 0.001). As shown in Table 4, the ObsQoR-11 scores at any measurement point were significantly associated with the EPDS scores and the weighted score of the 12-item WHODAS2.0 after adjusting for clinically relevant factors. The values of Cronbach’s coefficient of the EPDS at 1 month and 3 months and the WHODAS were 0.83, 0.74, and 0.87, respectively.

## 4. Discussion

We conducted a single-center prospective observational study to develop the ObsQoR-11J, assess its clinical utility, and investigate the association between the ObsQoR-11J score and the patient-reported outcome measures. The main finding of this study is that the ObsQoR-11J is a feasible, reliable, and valid tool for evaluating postoperative recovery. Lower ObsQoR-11J scores measured at any three postoperative points helped evaluate postpartum depression and functioning in patients who underwent cesarean delivery under spinal anesthesia.

Linguistic and cross-cultural translations of any instruments developed for health care research should follow established protocols, including obtaining permission from the original author and using an adequate sample size with at least 10 participants per questionnaire item [27]. Helping ensures that version validity and reliability are crucial to any global study, supporting the comparability of results obtained in different countries [28,29]. Regarding the sample size for cross-cultural translations of instruments, although Gorsuch (1983) described at least 50 respondents for a 10-item questionnaire [30], others have suggested a sample size of at least 10 participants for each scale item [31].

Previously, the ObsQoR has been translated into several different languages; however, several studies had small sample sizes and limited nulliparous women following scheduled cesarean delivery [6,22]. The ObsQoR-10, which is a short version of the ObsQoR-11, combines questions regarding postoperative pain (moderate and severe) into one item (pain) based on the patient’s request [32]. However, the QoR-15, which is a short version of the QoR-40 questionnaire used for non-obstetric populations, including older patients who are expected to have less comprehension than pregnant women, does not integrate moderate and severe pain [33]. The difficulty experienced by patients with this question has not been explained previously, although it may be related to time constraints. In addition, it remains unclear whether the ObsQoR-11 or ObsQoR-10 is more suitable for assessing postpartum recovery. Moreover, at the time this study was planned, ObsQoR-10 had not been developed, and only ObsQoR-11 was available as a measure of postpartum recovery.

This study included an adequate sample size and followed relevant protocols and standards established for scale development. Consequently, the completion rate, Cronbach’s alpha, ObsQoR-11 score at 24 h, correlation coefficient with the global health scale, and inter-item correlations were consistent with those previously reported (Table S4). This evidence suggests that the ObsQoR-11J is a feasible, reliable, and valid tool for assessing postoperative recovery after cesarean delivery. The feasibility of ObsQoR-11J was also demonstrated by the time required to complete the ObsQoR-11J, which was 67 ± 38 s, along with the high response rate (97.1%) and lack of inconvenience reported by the subjects. In this study, the correlation coefficient between the scores at 24 h and 25 h was not very high. The exact reason for this is unclear; however, these facts emphasize that when ObsQoR is measured the day after cesarean delivery in future studies, the exact time after surgery should be specified.

A recent study showed an association between poor early postpartum recovery and postpartum depression [13]; however, owing to the low prevalence of postpartum depression, the sample size required for multivariable logistic regression analysis was insufficient. In our study, multiple regression analysis with an adequate sample size (15 times the number of explanatory variables) showed similar results for postpartum depression and functioning, albeit with secondary outcomes. In addition, in contrast to a recent study in which preoperative anxiety and depression were assessed by self-report and medical records [13], our study used a valid tool, the Hospital Anxiety and Depression Scale, which has been used to assess mental health status in pregnant women [33,34,35].

In addition, the mean 12-item WHODAS2.0 score of the study population was 5.3 ± 8.1, and 86% of the participants had disability-free survival; however, one study conducted in South America showed a higher mean 12-item WHODAS2.0 score of 16.4 ± 16.0 [10]. The exact reason for these differences is unclear but may be related to attitudes surrounding pregnancy and access to medical care. Future studies should evaluate functional outcomes and depression in the postpartum population.

This study, using a self-report questionnaire, had several limitations. First, since our study was conducted at a Japanese facility with a longer postoperative hospital stay than in many Western countries, caution is required in generalizing and interpreting the results; however, we believe that a longer postoperative hospital stay led to a higher completion rate on postoperative day 5 [36]. Second, because researchers were unavailable, patient recruitment was limited and included cesarean deliveries that were predicted to begin after 8:30 am and end at 7:00 pm. Third, this study included cesarean deliveries performed under spinal anesthesia. The types of anesthesia—general, spinal, epidural, and combined spinal-epidural anesthesia—play a major role in postoperative recovery. Previous studies examining the effects of anesthesia on ObsQoR have involved a variety of anesthesia methods, making interpretation difficult [37,38]. However, this study was limited to spinal anesthesia, the most common type, which is both a limitation and a strength of this research.

Fourth, the 12-item WHODAS2.0 was not assessed at one month. As mentioned above, the hospitalization period after cesarean delivery is longer in Japan, and evaluations conducted one month after the operation date include the condition during the hospitalization period; thus, we did not evaluate the WHODAS2.0. Fifth, although the response rate for outcomes at three months was high (approximately 84%), the reasons for non-response were not assessed, and the possibility that patients in poor condition may not have responded must be considered. Sixth, we did not perform a confirmatory factor analysis. Finally, in this study, we analyzed the effect of immediate postpartum recovery on medium-term functional disability and mental health, adjusting for clinically relevant factors. However, because external influences such as pandemic restrictions, social isolation, and medical uncertainty can impact the medium-term health status of women [39,40,41], future studies should evaluate these factors. Finally, this study did not conduct a survey for more than 3 months.

In contrast, the strengths of our study were that ObsQoR-11 was used in the early and intermediate postoperative periods, and postpartum depression and functioning were assessed three months after delivery. Furthermore, we believe that demonstrating this association would be beneficial for both healthcare providers and patients. Although postpartum depression has been investigated for many years, the assessment of functioning has been insufficient, and further studies are needed. Although the ObsQoR has been translated into several languages, no Japanese version is available. Another strength of this study is that it developed a tool that is valid and reliable for patients whose native language is Japanese, as it can be adapted to consider cultural backgrounds and linguistic differences.

In summary, we translated the ObsQoR-11 into Japanese, assessed its usefulness in a clinical setting, and found that lower ObsQoR-11 scores were associated with postpartum depression and non-disability-free survival. This study highlights the potential contribution of the ObsQoR as an early predictor of postpartum depression and non-disability-free survival.

## Figures and Tables

**Table 1:** Patient characteristics.

	Total (*n* = 137)
Age (year), mean (SD)	34.0 (5.5)
Height (cm), mean (SD)	157.6 (4.9)
Weight (kg), mean (SD)	64.7 (11.0)
Drinking habits, number (%)	8 (5.8)
Smoking, number (%)	1 (0.7)
Gestational age (weeks), mean (SD)	37.2 (3.1)
Presence of partner, number (%)	129 (94.2)
Previous cesarean section, number (%)	
0	74 (54.0)
1	49 (35.8)
2	14 (10.2)
Treatment for infertility, number (%)	40 (28.9)
Gravidity (number), mean (SD)	2.1 (1.2)
HADS score	
Anxiety score, mean (SD)	5.7 (3.5)
Number of patients with anxiety score ≥ 8, number (%)	41 (29.9)
Depression score, mean (SD)	5.3 (3.2)
Number of patients with depression score ≥ 8, number (%)	36 (26.2)
Number of fetuses, number (%)	
Single	115 (83.9)
Twin	22 (16.1)
Hypertensive disorders of pregnancy, number (%)	13 (9.5)
Gestational diabetes, number (%)	8 (5.8)
Thyroid disease, number (%)	10 (7.4)
Ritodrine administration, number (%)	24 (17.5)
Magnesium infusion, number (%)	28 (20.4)
Urgent surgery, number (%)	57 (41.6)
Surgical duration (min), mean (SD)	60.9 (18.8)
Intraoperative blood loss (mL), mean (SD)	594 (342)

HADS: Hospital Anxiety and Depression Scale. SD: standard deviation.

**Table 2:** Obstetric Quality of Recovery-11 (ObsQoR-11J) scores 24 h after cesarean section.

ObsQoR-11J Item Number	Mean (Standard Deviation)	Minimum	Maximum	Floor Effect (%)	Ceiling Effect (%)
1	5.1 (1.9)	0	10	2.1	0.7
2	6.2 (2.6)	0	10	2.1	10.9
3	8.4 (2.7)	0	10	2.1	60.5
4	9.1 (1.6)	3	10	2.1	68.6
5	9.1 (2.0)	0	10	1.4	75.1
6	5.3 (2.8)	0	10	12.4	5.8
7	5.9 (3.3)	0	10	10.9	22.6
8	4.5 (4.1)	0	10	32.8	24.8
9	3.0 (3.5)	0	10	47.5	7.3
10	5.0 (3.8)	0	10	24	21.1
11	5.2 (2.8)	0	10	9.4	7.3
Total	67.2 (19.2)	25	106	0	0

**Table 3:** Secondary outcomes after cesarean section.

	Mean (SD) or Number (%)
EPDS at 1 month (*n* = 130), mean (SD)	4.5 (3.9)
Positive screening for postpartum depression at 1 month (*n* = 130), number (%)	21 (16.1)
EPDS at 3 months (*n* = 115), mean (SD)	2.8 (2.7)
Positive screening for postpartum depression at 3 months (*n* = 115), number (%)	5 (4.3)
Weighted score of 12-item WHODAS2.0 at 3 months (*n* = 116), mean (SD)	5.3 (8.1)
DFS at 3 months (*n* = 116), number (%)	100 (86.3)

EPDS, Edinburgh Postnatal Depression Scale; WHODAS2.0, World Health Organization Disability Assessment Schedule 2.0; DFS, disability-free survival; SD, standard deviation.

**Table 4:** Adjusted standardized coefficient of the ObsQoR-11 score and EPDS at 1 and 3 months and a weighted score of the 12-item WHODAS at three months.

	EPDS at 1 Month		EPDS at 3 Months	Weighted Score of 12-Item WHODAS2.0 at 3 Months		
	Adjusted Standardized Coefficient (95% CI)	*p* Value	Adjusted Standardized Coefficient (95% CI)	*p* Value	Adjusted Standardized Coefficient (95% CI)	*p* Value
ObsQoR-11 at 24 h after surgery	−0.23 (−0.08, −0.01)	0.006	−0.25 (−0.06, −0.01)	0.004	−0.20 (−0.17, −0.02)	0.012
ObsQoR-11 on postoperative day 3	−0.29 (−0.16, −0.04)	0.001	−0.20 (−0.10, −0.007)	0.02	−0.17 (−0.27, −0.001)	0.042
ObsQoR-11 on postoperative day 5	−0.31 (−0.18, −0.05)	<0.001	−0.27 (−0.12, −0.02)	0.003	−0.22 (−0.32, −0.04)	0.011

ObsQoR-11, Obstetric Quality of Recovery-11; CI, confidence interval; EPDS, Edinburgh Postnatal Depression Scale; WHODAS2.0, WHO Disability Assessment Schedule 2.0. Age, urgency, presence of a partner, infertility treatment history, preoperative anxiety or depression, and surgical duration were adjusted.

## Data Availability

Data will be provided upon reasonable request.

## Supplementary Materials

The following supporting information can be downloaded at https://www.mdpi.com/article/10.3390/jcm14041390/s1, Figure S1: The ObsQoR-11 score 24 h after cesarean delivery showed a normal distribution; Figure S2. Obstetric quality of recovery-11 score 24 h after cesarean section; Table S1: Inter-item correlation matrix for the Obstetric Quality of Recovery-11 assessed 24 h after cesarean section; Table S2: Results of the linear mixed model, treating timepoints as categorical data; Table S3: Correlation matrix among ObsQoR-11, EPDS, and 12-item WHODAS; Table S4: Comparison among previous reports.

## Abbreviations

The following abbreviations are used in this manuscript:

ObsQoR-11	Obstetric Quality of Recovery-11 questionnaire
ObsQoR-11J	Japanese version of Obstetric Quality of Recovery-11 questionnaire
EPDS	Edinburgh Postnatal Depression Scale
WHODAS	World Health Organization Disability Assessment Schedule
EQ-5D-3L	EuroQol 5 dimensions 3-level
DFS	Disease-free survival
