# Supplementary material for: Validation of the Japanese Version of Obstetric Quality of Recovery-11 Questionnaire and Its Association with Postpartum Depression and Functional Outcomes: A Prospective Observational Study
Source: J Clin Med. 2025 Feb 19;14(4):1390. doi: 10.3390/jcm14041390 (PMC11855944; doi:10.3390/jcm14041390)
Supplement: Supplementary file 1 [file jcm-14-01390-s001.zip › jcm-3478859-supplementary.pdf]

Journal of
Clinical Medicine

MDPI

Supplementary Material

Table S1. Inter-item correlation matrix for the Obstetric Quality of Recovery-11 assessed 24 h after cesarean section.

Item		1	2	3	4	5	6	7	8	9	10	11	Total
1	correlation coefficient	1											
	P value												
2	correlation coefficient	0.76	1										
	P value	<0.001											
3	correlation coefficient	0.08	0.03	1									
	P value	0.32	0.69										
4	correlation coefficient	0.22	0.29	0.44	1								
	P value	0.009	0.001	<0.001									
5	correlation coefficient	0.22	0.22	0.22	0.23	1							
	P value	0.01	0.01	0.01	0.006								
6	correlation coefficient	0.53	0.48	0.15	0.33	0.23	1						
	P value	<0.001	<0.001	0.06	<0.001	0.008							
7	correlation coefficient	0.23	0.19	-0.01	0.14	-0.06	0.31	1					
	P value	0.007	0.02	0.9	0.09	0.48	<0.001						
8	correlation coefficient	0.06	0.06	0.03	0.01	-0.12	0.18	0.55	1				
	P value	0.45	0.44	0.66	0.83	0.18	0.03	<0.001					
9	correlation coefficient	0.01	0.03	-0.01	0.03	-0.10	0.16	0.52	0.76	1			
	P value	0.84	0.67	0.83	0.73	0.23	0.05	<0.001	<0.001				
10	correlation coefficient	0.18	0.135	0.05	0.12	-0.15	0.19	0.70	0.54	0.54	1		
	P value	0.02	0.12	0.54	0.13	0.07	0.02	<0.001	<0.001	<0.001			
11	correlation coefficient	0.30	0.31	0.10	0.23	0.12	0.48	0.48	0.30	0.35	0.47	1	
	P value	<0.001	<0.001	0.16	0.007	0.14	<0.001	<0.001	<0.001	<0.001	<0.001		
Total	correlation coefficient	0.50	0.49	0.29	0.40	0.18	0.59	0.74	0.68	0.66	0.71	0.67	1
	P value	<0.001	<0.001	<0.001	<0.001	0.03	<0.001	<0.001	<0.001	<0.001	<0.001	<0.001	

Table S2. Results of the linear mixed model, treating timepoints as categorical data.

	Estimate	Standard error	*P* value
24 h after surgery	66.9	1.2	
Postoperative day 3	21.9	1.2	< 0.001
Postoperative day 5	29.1	1.3	< 0.001

Table S3. Correlation matrix among ObsQoR-11, EPDS, and 12-item WHODAS.

		ObsQoR-11 at 24 h after surgery	ObsQoR-11 on postoperative day 3	ObsQoR-11 on postoperative day 5	EPDS at 1 month after surgery	Weighted score of 12-item WHODAS	EPDS at 3 months after surgery
ObsQoR-11 at 24 h after surgery	correlation coefficient	1					
	P value						
	number	134					
ObsQoR-11 on postoperative day 3	correlation coefficient	0.4	1				
	P value	< 0.001					
	number	130	133				
ObsQoR-11 on postoperative day 5	correlation coefficient	0.36	0.64	1			
	P value	< 0.001	< 0.001				
	number	124	125	127			

 https://doi.org/10.3390/jcm14041390

EPDS at 1 month after surgery	correlation coefficient	-0.2	-0.26	-0.25	1		
	P value	0.01	0.003	0.004			
	number	127	128	125	130		
Weighted score of 12-item WHODAS	correlation coefficient	-0.27	-0.21	-0.33	0.33	1	
	P value	0.003	0.02	< 0.001	< 0.001		
	number	113	115	113	115	116	
EPDS at 3 months after surgery	correlation coefficient	-0.25	-0.23	-0.31	0.56	0.43	1
	P value	0.006	0.01	0.001	< 0.001	< 0.001	
	number	112	114	113	114	112	115

ObsQoR-11: Obstetric Quality of Recovery-11; EPDS: Edinburgh Postnatal Depression Scale, WHODAS 2.0, World Health Organization Disability Assessment Schedule 2.0.

Table S4. Comparison among previous reports.

**Author (year)**	**Language**	**Recruitment rate**	**Completion rates at 24 h**	**ObsQoR-11 score at 24 h**	**Cronbach alpha**	**Correlation coefficient#**	**Interitem correlations**
Kang et al. [1] (1)	Korean	100% (120/120)	100% (120/120)	61.3 (16.2)	0.78	0.73	-0.01, 0.79
Chou et al. [2] (2)	Chinese	NA	100% (279/279)	NA	0.75	0.38	NA
Gupta et al. [3] (3)	Hindi	NA	90.0% (108/120)	75.9 (4.0)	0.91	0.80	NA
Ozkan et al. [4] (4)	Turkish	94.0% (191/203)	97.3% (186/191)	NA	0.82	0.85	-0.17, 0.69
Ishida et al. (this study)	Japanese	92.0% (138/150)	97.1% (134/138)	67.2 (19.2)	0.77	0.43	-0.15, 0.76

NA, not available, ObsQoR-11, Obstetric Quality of Recovery-11. #Correlation coefficient between the scores of the ObsQoR-11J and general health scale, including the visual analog scale or numerical rating scale.

References

1. Kang, R.; Lee, S.; Lee, E.; Cho, Y.J.; Jeong, J.S.; Choi, S.J.; Gwak, M.S.; Sim, W.S.; Kim, D.K.; Ko, J.S. Validation and clinical utility of the Korean version of the obstetric quality-of-recovery score (ObsQoR-11) following elective Cesarean section: A prospective observational cohort study. *Diagnostics (Basel, Switzerland)* **2022**, *12*, 291. DOI:10.3390/diagnostics12020291.
2. Chou, W.H.; Lee, S.O.; Sun, M.H.; Tseng, Y.C.; Chan, K.C.; Chen, Y.H.; Wu, C.Y. Validation of Chinese version of a global anesthetic recovery questionnaire: A multicenter observational trial on ObsQoR-11. *J Formos Med Assoc Taiwan Yi Zhi* **2023**, *122*, 479–485. DOI:10.1016/j.jfma.2022.12.010.
3. Gupta, S.; Choudhary, S.; Choudhary, V.; Jain, K.; Bhatia, N.; Gupta, A. Validation of Hindi version of the obstetric quality of recovery score-11 (ObsQoR-11 H) following elective caesarean section. *Indian J Anaesth* **2023**, *67* Suppl 4, S251–S256. DOI:10.4103/ija.ija_69_23.
4. Ozkan, G.; Kara, U.; Ince, M.E.; Ozdemir, O.; Ulubay, M.; Senkal, S. Validation of the Turkish version of the Obstetric Quality-of-Recovery score 11 (ObsQoR-11T) after cesarean delivery. *Health Qual Life Outcomes* **2022**, *20*, 155. DOI:10.1186/s12955-022-02073-y.

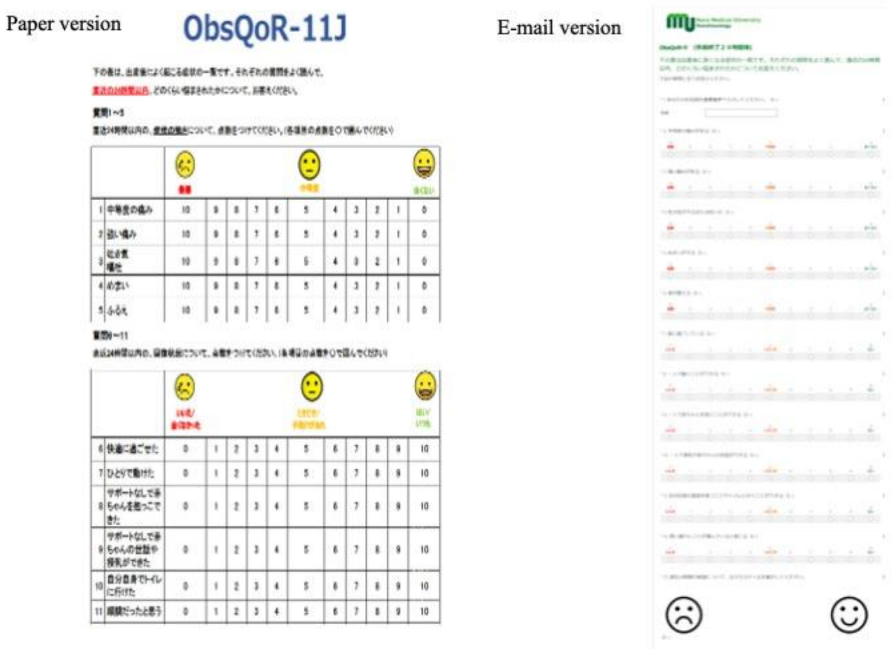

Figure S1. The ObsQoR-11 score 24 h after cesarean delivery showed a normal distribution.

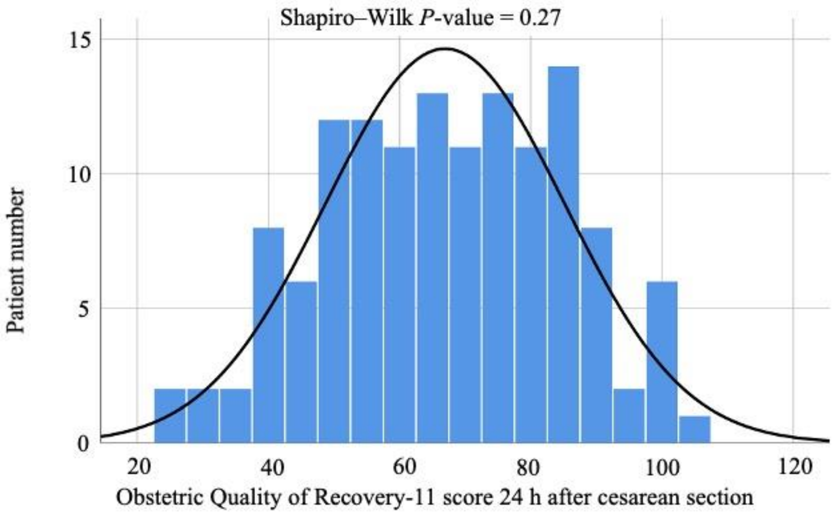

Figure S2. Obstetric quality of recovery-11 score 24 h after cesarean section.
